# Editorial: Visualizing plant-microbe dynamics: cutting-edge imaging methods in plant research

**DOI:** 10.3389/fpls.2025.1653745

**Published:** 2025-07-08

**Authors:** Ankush Prasad, Claudio Rossi, Pavel Pospíšil

**Affiliations:** ^1^ Department of Biophysics, Faculty of Science, Palacký University, Olomouc, Czechia; ^2^ Department of Biotechnology, Chemistry and Pharmacy, University of Siena, Siena, Italy

**Keywords:** plant-microbe interactions, imaging techniques, plant physiology, rhizosphere, arbuscular mycorrhizal fungi

Understanding the complex interactions between plants and microbes is vital for advancing sustainable agriculture and improving plant health. Recent developments in technologies have revolutionized our ability to observe these interactions in real time and at high precision. Techniques such as stereoscopic and confocal microscope, *in situ* hybridization (FISH), and gene expression analysis allow researchers to understand microbial colonization, biofilm formation, and plant immune responses. Advanced OMICS and imaging methods are also being integrated with molecular tools and computational modelling to map spatial-temporal dynamics in the plant-microbe dynamics studies. These innovations are not only deepening our knowledge of plant-microbe relationships but also opening new avenues for targeted microbial interventions in crop systems. With the increasing urgency of climate change, food security, and sustainable farming practices, the need to unravel these interactions has never been more pressing. The Research Topic *“Visualizing Plant-Microbe Dynamics: Cutting-Edge Imaging Methods in Plant Research”* aims to spotlight the transformative potential of modern technologies in deepening our understanding of the complex interaction between plant roots and their microbial allies.

This Research Topic brings together innovative studies that apply microscopic imaging, *in vitro* symbiosis models, and other state-of-the-art methodologies to decipher plant-microbe associations. Among the notable contributions, the work by Liu et al. titled *“Exogenous myristate promotes the colonization of arbuscular mycorrhizal fungi in tomato”* stands out for its integration of symbiosis assessment with functional lipid supplementation experiments. Liu et al. explore the fascinating ability of arbuscular mycorrhizal fungi (AMF) to enhance root colonization under the influence of exogenously applied myristate. Utilizing dual *in vitro* culture systems and sand-based growth models, the authors provide a visually and quantitatively rich portrayal of AMF development, from hyphal branching and hyphopodia formation to intraradical structures. Their accurate observations show that myristate significantly boosts hyphal proliferation and the frequency of arbuscule and vesicle formation. This study highlights the pivotal role of environmental lipids in symbiotic signalling and colonization, made visible through precise measurements and root colonization metrics. Equally significant is the meta-analysis by Yuan et al., who evaluated the role of AMF in enhancing secondary metabolite production in medicinal plants. Though not imaging-focused per se, their findings emphasize the functional output of symbiosis and the enhanced level of active ingredients in medicinal plants.

In the study presented by Jiang et al., the authors shed light on the intricate interplay between soil microbiota and nutrient dynamics in iron-deficient citrus orchards on coastal saline-alkali lands. The authors demonstrate that citrus roots significantly influence the rhizosphere microenvironment by nurturing beneficial bacterial communities capable of enhancing iron availability and mitigating salt stress. By employing 16S rRNA amplicon sequencing and advanced ecological network analyses, they showed a distinct stratification in bacterial composition and function between rhizosphere and bulk soils, linked closely to variations in nutrient content and salinity with soil depth.

**Figure 1:**
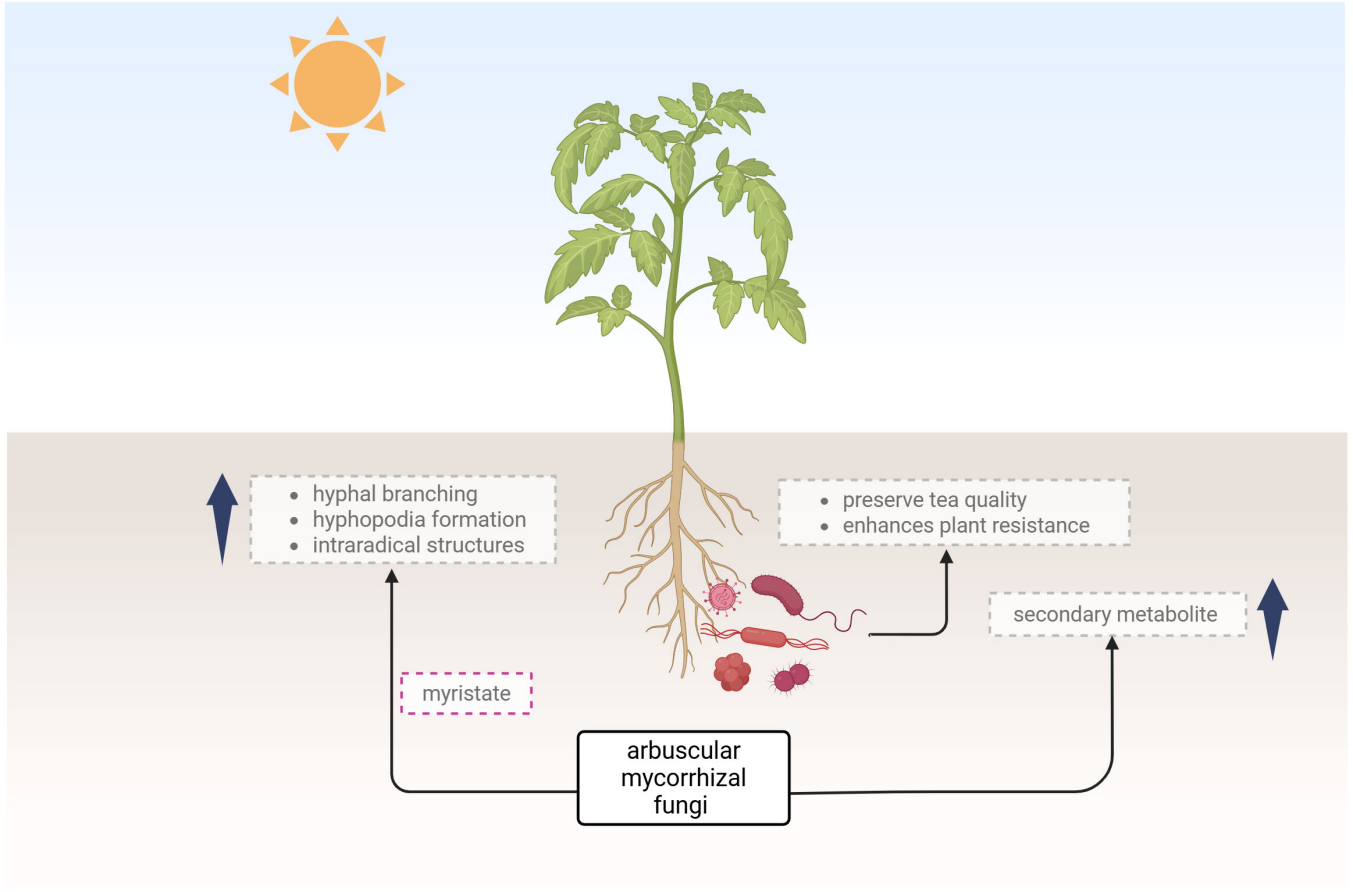
The illustration depicts the influence of arbuscular mycorrhizal fungi and microbial communities on plant development, highlighting their role in enhancing plant resistance and secondary metabolite production created using Biorender.com.

In the review by Xie et al., the authors highlight the importance of microbial stability in tea plantations for preserving tea quality and plant health. Microbial communities impact tea flavour and help plants resist pests and diseases. While biotic stress can harm plants, it also triggers defence mechanisms and microbial changes. Microbial biocontrol agents (MBCAs) offer a natural alternative to pesticides, controlling pests and shaping beneficial microbial communities. Although research on MBCAs is promising, their real-world impact in tea fields is poorly understood. The review explores plant-insect-microbe interactions to guide better pest and disease management in tea cultivation (**Figure 1**).

In an era of precision agriculture and ecological restoration, the ability to visualize these interactions *in situ* is more than a technical achievement; it is a pathway to more resilient and sustainable ecosystems. We invite readers to explore this Research Topic, which centres on the convergence of molecular biology and advanced imaging technologies.

